# The roles and mechanisms of the NF-κB signaling pathway in tendon disorders

**DOI:** 10.3389/fvets.2024.1382239

**Published:** 2024-06-24

**Authors:** Hanyue Li, Yini Li, Shengyu Luo, Yan Zhang, Zhenhua Feng, Sen Li

**Affiliations:** ^1^School of Physical Education, Southwest Medical University, Luzhou, China; ^2^Department of Ultrasound, Affiliated Hospital of Southwest Medical University, Luzhou, Sichuan, China; ^3^Luzhou Vocational and Technical College, Luzhou, China; ^4^Division of Spine Surgery, Department of Orthopedic Surgery, Affiliated Hospital of Medical School, Nanjing Drum Tower Hospital, Nanjing, Jiangsu, China

**Keywords:** NF-κB signaling pathway, tendon injury, tendon scar healing, tendon adhesion tendinopathy, tendon repair

## Abstract

Both acute and chronic tendon injuries are the most frequently occurring musculoskeletal diseases in human and veterinary medicine, with a limited repertoire of successful and evidenced-based therapeutic strategies. Inflammation has been suggested as a key driver for the formation of scar and adhesion tissue following tendon acute injury, as well as pathological alternations of degenerative tendinopathy. However, prior efforts to completely block this inflammatory process have yet to be largely successful. Recent investigations have indicated that a more precise targeted approach for modulating inflammation is critical to improve outcomes. The nuclear factor-kappaB (NF-κB) is a typical proinflammatory signal transduction pathway identified as a key factor leading to tendon disorders. Therefore, a comprehensive understanding of the mechanism or regulation of NF-κB in tendon disorders will aid in developing targeted therapeutic strategies for human and veterinary tendon disorders. In this review, we discuss what is currently known about molecular components and structures of basal NF-κB proteins and two activation pathways: the canonical activation pathway and the non-canonical activation pathway. Furthermore, we summarize the underlying mechanisms of the NF-κB signaling pathway in fibrosis and adhesion after acute tendon injury, as well as pathological changes of degenerative tendinopathy in all species and highlight the effect of targeting this signaling pathway in tendon disorders. However, to gain a comprehensive understanding of its mechanisms underlying tendon disorders, further investigations are required. In the future, extensive scientific examinations are warranted to full characterize the NF-κB, the exact mechanisms of action, and translate findings into clinical human and veterinary practice.

## 1 Introduction

Both acute and chronic tendon injuries represent the most frequent musculoskeletal ailments in human and veterinary medicine (1). In humans, an estimated 30% of musculoskeletal consultations are tendon disorders (2). In veterinary medicine, approximately 50% of tendon disorders influence racehorses, leading to a long shadow of financial losses due to compromised sports performances (3). However, tendon injuries in all species are challenging as their limited regenerative ability makes tendon repair ineffective, resulting in the formation of inferior scar tissue (4, 5). Moreover, scar-mediated healing is particularly problematic in the flexor tendon as excessive scar tissue can form adhesion between the tendon, sheath, and surrounding tissue, further disrupting tendon function (6). On the other hand, the accumulation of microdamage during overloading after unsuccessful tendon regenerative healing is a predisposing factor for the progression of degenerative tendinopathy (7). Degenerative tendinopathy is a broad spectrum of chronic tendon disorders characterized by swelling, pain, ossification, and physical disability (8). Despite the prevalence of these diseases, current therapeutic strategies often fall short of achieving complete functional recovery of the tendon (9). Therefore, to exploit novel therapeutic strategies for enhancing tendon regenerative healing and preventing the progression of degenerative tendinopathy, it is pivotal to understand the basic mechanisms underlying tendon pathologies and scar healing (10).

The significance of inflammation in both the tendon scar healing and the early initiation of degenerative tendinopathy has been highlighted in recent years in patient samples in human and veterinary species (11, 12). To alleviate the inflammation of tendon disorders, anti-inflammatory therapeutic approaches such as non-steroidal anti-inflammatory drugs (NSAIDs) are commonly prescribed to treat tendon disorders (13, 14). However, these therapeutic interventions to completely block the inflammation have been explored with mixed success, possibly because controlled inflammation is beneficial for tendon repair (15). Toward this end, recent investigations have indicated that a more precise targeted approach for modulating inflammation is better than completely blocking it in humans and equines (16).

The NF-κB signaling pathway has been regarded as a typical proinflammatory signal transduction pathway, the activation of which is involved in the regulation of multiple genes, including proinflammatory cytokines, chemokines, adhesion molecules, and enzymes (17, 18). It has been reported that blocking the activation of NF-κB signaling pathway in tendon disorders has been more effective than completely inhibiting all the inflammatory response (19–21). Nevertheless, there is currently insufficient mechanistic understanding of the NF-κB signaling pathway in tendon disorders to manifest this possibility in practice. In this biomedical challenge, rodent models of induced tendon disorders and *in vitro* cell/tissue culture reports function as preclinical models to obtain critical biological information and as translational models to drive advancements in human and veterinary medicine (22). While the prevalence and importance of tendon disorders vary between species, most of the mechanisms triggering their progression are alike. This suggests that we can learn about the NF-κB signaling pathway in tendon disorders by both using animal data to improve human studies and using human data in animal research. Here, in this review, we provide a summary of our current understanding of the mechanisms of the NF-κB signaling pathway underlying the degenerative tendinopathy and tendon scar healing in both species and highlight the effect of targeting this signaling pathway in tendon disorders, which can give some indications for veterinary clinical application. Nevertheless, future investigations are still warranted to investigate the exact mechanisms of action.

## 2 NF-κB “identity card”

### 2.1 Molecular components and structures of the NF-κB family

Transcription factors are proteins responsible for gene expression through binding to gene enhancer and promoter sites (23). NF-κB is a family of transcription factors expressed in almost all cells and participates in the regulation of various cell processes and signaling pathways (24). To date, NF-κB was first discovered as a transcription modulator in 1986, which binds to the intrinsic enhancer of the kappa light chain gene (the κB site) in B lymphocytes (25, 26). Subsequently, NF-κB has been found to be one of the highly conserved protein families, present in many organisms, such as humans, mice, and equines (27).

**Figure 1:**
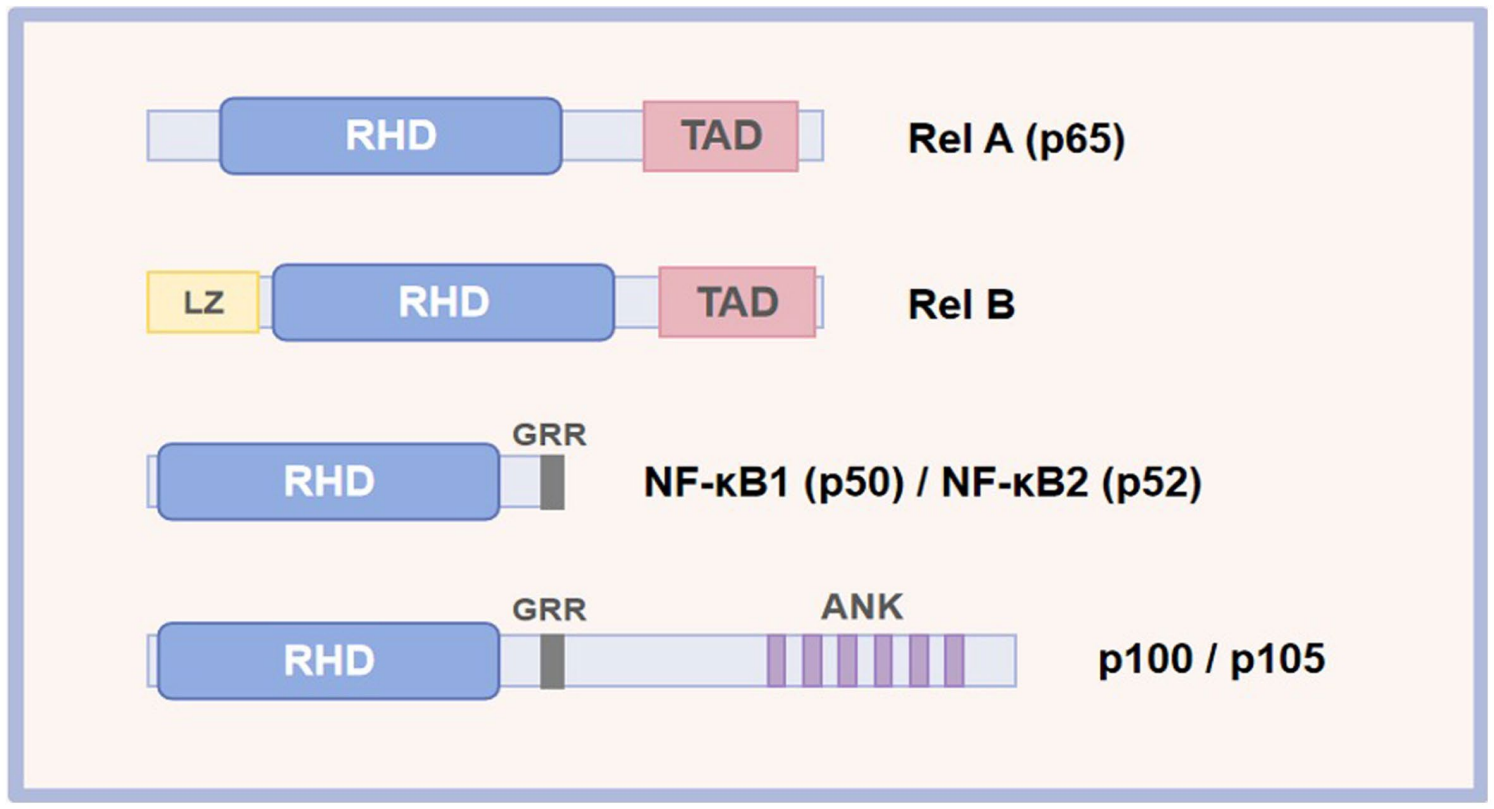
Schematic representation of the NF-κB protein family. RHD, rel homology; TAD, transcriptional activation domain; LZ, leucine zipper; ANK, ankyrin repeat; GRR, glycine-rich repeat.

The mammalian NF-κB represents a family of transcription factors, mainly including RelA (p65), c-Rel, RelB, NF-κB1 (p50), and NF-κB2 (p52; Table 1) (28). These proteins interact with each other to form dimers that can bind to DNA and activate transcription (Figure 1). These members are split into two groups according to their manner of inactivation and structural similarities. The first group contains RelA, c-Rel, and RelB, all of which are characterized by the presence of a C-terminal transcription activation domain (TAD) and an N-terminal rel homology domain (RHD) of the protein structure (29, 30). TAD of the protein structure is required for NF-κB dimer translocation to the nucleus, while RHD within these proteins serves to bind with the inhibitory Kappa B (IκB) proteins to retain NF-κB in the cytoplasm (31). Inconsistent with other members, RelB does not homodimerize, and it is characterized by the presence of a leucine zipper (LZ) motif in its N terminus, which exerts a critical role in gene transcription (28). Nevertheless, it remains to be determined whether the LZ confers other functional specificity to RelB, including heterodimerization with NF-κB2 or another possible coagent. Differently, another group, NF-κB1 and NF-κB2, lack TAD. Thus, NF-κB1 and NF-κB2 homodimers cannot activate gene transcription or act as a transcriptional repressor, but they are synthesized as large inactive precursors p105 and p100 that yield mature NF-κB subunits p50 and p52, respectively (32, 33). The ankyrin repeats (ANK) in the C-terminal region of p105 and p100 participate in their degradation along the ubiquitin-proteasome pathway (34).

**Table 1:** The NF-κB transcription factors.

Protein	Human gene	Function
p105/p50	NFKB1	Canonical signaling pathway
p100/p52	NFKB2	Non-canonical signaling pathway
p65	RELA	Canonical signaling pathway
RelB	RELB	Gene transcription
c-Rel	REL	Contains TAD

### 2.2 General function and regulation of NF-κB

The activation of NF-κB after responding to an external or internal stimulus occurs in two main ways: the canonical and noncanonical (alternative) pathways (35). The canonical pathway will predominately result in the activation of RelA (p65), NF-κB1 (p50), and c-Rel dimers, while the alternative pathway is primarily responsible for the activation of NF-κB2 (p52) and RelB dimers (36). However, it is worth noting that these two signaling pathways are not mutually exclusive. For instance, the canonical p65/p50 dimers have been reported to be involved in the regulation of the alternative pathway (26).

The IκB proteins and IκB kinase (IKK) complex tightly regulate the activation of the NF-κB signaling pathway (37, 38). IκB is a specific inhibitor of NF-κB. The IκB proteins in mammalian contain seven members: IκBα, IκBβ, IκBε, IκBδ, Bcl-3, IκBξ, and IκBNS (Table 2) (39). They block the nucleus localization signal by binding to the RHD region of NF-κB, therefore preventing NF-κB from entering the nucleus to perform its function. The IKK complex consisting of two subunits, IKKα (IKK1) and IKKβ (IKK2), and one regulatory subunit, IKKγ (NEMO), is responsible for the degradation of the IκB proteins, allowing NF-κB proteins free to translocate in the nucleus where it binds to the promoter region of the target genes (40, 41). Moreover, IKKα is primarily responsible for the activation of the noncanonical pathway by phosphorylating and processing of p100. In contrast, IKKβ and IKKγ are required for the ignition of the canonical pathway by phosphorylating of IκB proteins (42).

**Table 2:** The IκΒ family.

Protein	Function
IKBα	Inhibiting of the canonical NF-κB activation
IKBβ	Inhibiting of the canonical NF-κB activation
	Prolonged the activation of NF-κB signaling pathway
IKBδ (p100)	Inhibiting of the noncanonical NF-κB activation
IKBε	Inhibiting of the canonical NF-κB activation
	Negative regulation of NF-κB response
Bcl-3	Transcriptional co-repressor, specifically regulating TNFα
IκBNS	Transcriptional co-repressor, specifically regulating IL-6
IκBξ	Transactivation of IL-6
	Inhibitor of NF-κB activity

The canonical signaling pathway is primarily responsible for sensing tissue damage, infection, and proinflammatory signals (43). After tissue injury, this pathway is activated by various proinflammatory signals (cytokine receptors such as IL-1R and TNF-R), toll-like receptors (TLR), T cell receptors (TCR), and B cell receptors (BCR) (44). When an infection or proinflammatory signals arrive in the cell, the receptors mentioned above can be activated. Activation of these receptors induces the activation of IKK, and then the activated IKK triggers the phosphorylation of IκB, leading to its ubiquitination and subsequent proteasomal degradation. The degradation of IκB proteins unmasks a nuclear localization signal, offering NF-κB dimers entrance ability to the nucleus. This process further accelerates the transcription of various proinflammatory cytokines, proteinases, and prostaglandins, contributing to the activation of inflammatory cells (macrophages and neutrophils) (45). On the other hand, the alternative signaling pathway of NF-κB is primarily involved in lymphoid organogenesis and the activation of the adaptive immune response (46). In contrast to the canonical NF-κB signaling pathway, the alternative signaling pathway is activated by a subset of Tumor Necrosis Factor Receptor (TNFR) superfamily members, including CD40, the lymphotoxin-β receptor (LTβR), receptor activator of NF-κB ligand (RANK), and B lymphocyte stimulating factor receptor (BAFFR) (47). Furthermore, this alternative pathway of NF-κB activation depends on the inducible processing of p100 precursor protein rather than the degradation of IκB proteins of the canonical pathway. The C-terminal ARD domain of p100 functions like IκB proteins to keep NF-κB dimers in an inactive state (48). Its C-terminal part owes its inhibitory effect to the presence of ankyrin repeats-33 amino acid residue motifs, which resemble those present in IκB proteins and are responsible for binding to NF-κB proteins (49, 50). In general, stimulation from these receptors leads to the activation of the NIK protein, which further induces the phosphorylation of the IKKα and promotes the degradation of p100 to generate p52. Following the phosphorylation cascade, the p52 subunit dimerizes RelB, resulting in the expression of NF-κB target genes (51).

**Figure 2:**
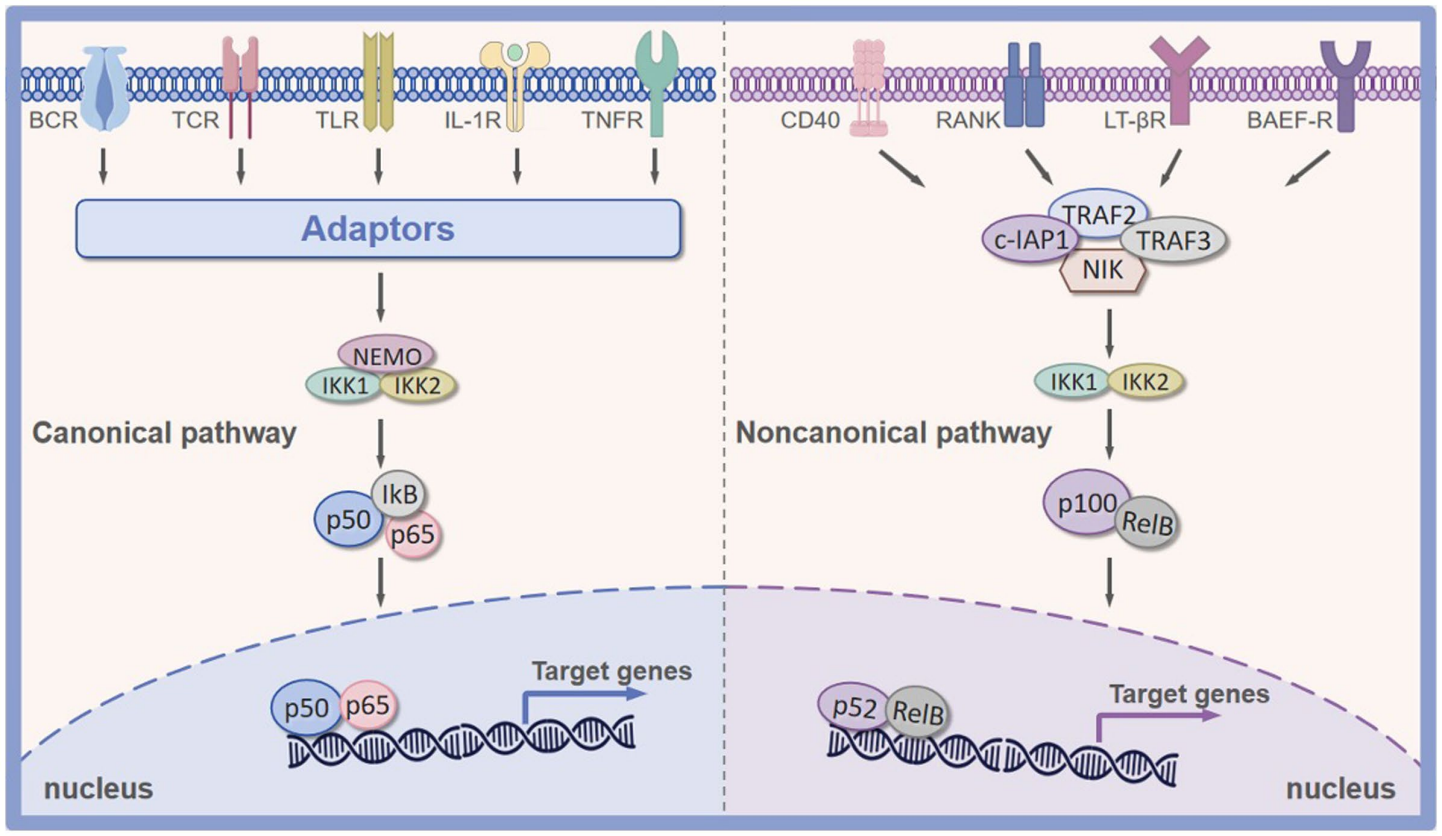
Canonical and noncanonical pathway of NF-κB signaling pathway. Canonical NF-κB signaling pathway is primarily activated by TNFR, IL-1R, TLR, BCR, and TCR. Non-canonical NF-κB signaling pathway is predominated activated by CD40, RANK, LTβR, and BAFFR. BAFFR, B lymphocyte stimulating factor receptor; RANK, receptor activator of nuclear factor kappa-B ligand; TLR, Toll-like receptor; IL-1R, interleukin-1 receptor; TNFR, tumor necrosis factor receptor; LTβR, lymphotoxin β-receptor; BCR, B-cell receptor; TCR, T-cell receptor; CD40, CD40 ligand receptor.

Overall, these aspects of NF-κB function and activation are undoubtedly critical to our understanding of this family of NF-κB’s overall behavior. They also provide a foundation for therapeutic targeting in tendon disorders based on NF-κB inhibition (Figure 2).

## 3 Fibrotic-scarring tendon healing, adhesion, and degenerative tendinopathy

In a manner similar to other tissue, tendon healing in veterinary species also consists of three distinct but overlapping phases: (1) the inflammatory phase (a few days), (2) the proliferative phase (several weeks), (3) the remodeling phase (lasting months/years) (52). In the inflammatory phase, the injury site is predominately infiltrated by macrophages. They produce a variety of proinflammatory cytokines, such as TNF-α and IL-1β, that amplify inflammatory response, leading to the activation of other immune cells and activation of tenocytes and tendon stem/progenitor cells (TSPCs) (53). Following this, the proliferative phase is featured by the expansion of ECM, increased cellularity, and depositional of the fibrovascular scar by tenocytes. About 2 weeks after injury, remodeling the injured area during the remodeling phase starts with the reorganization of the newly deposited collagen, with a gradual decrease in cellularity and an increase in the fibrous matrix (54). However, despite efforts to repair, the ensuing healing processes often fail to yield fully regenerative healing of the injured tissue, resulting in biomechanically inferior scar tissue formation (55). Moreover, the proliferation of scar tissue between the tendon and adjacent tissues is also undesirable as this can lead to the adhesion formation, further impeding normal tendon gliding and function (56). If the tendon continues to undergo microdamage after unsuccessful tendon regenerative healing, the local cells in tendon tissue undergo pathological alternations, resulting in the destruction of tendon tissue. This can progress to the degenerative tendinopathy (57).

## 4 The mechanisms of NF-κB in tendon fibrotic scar healing, adhesion

**Figure 3:**
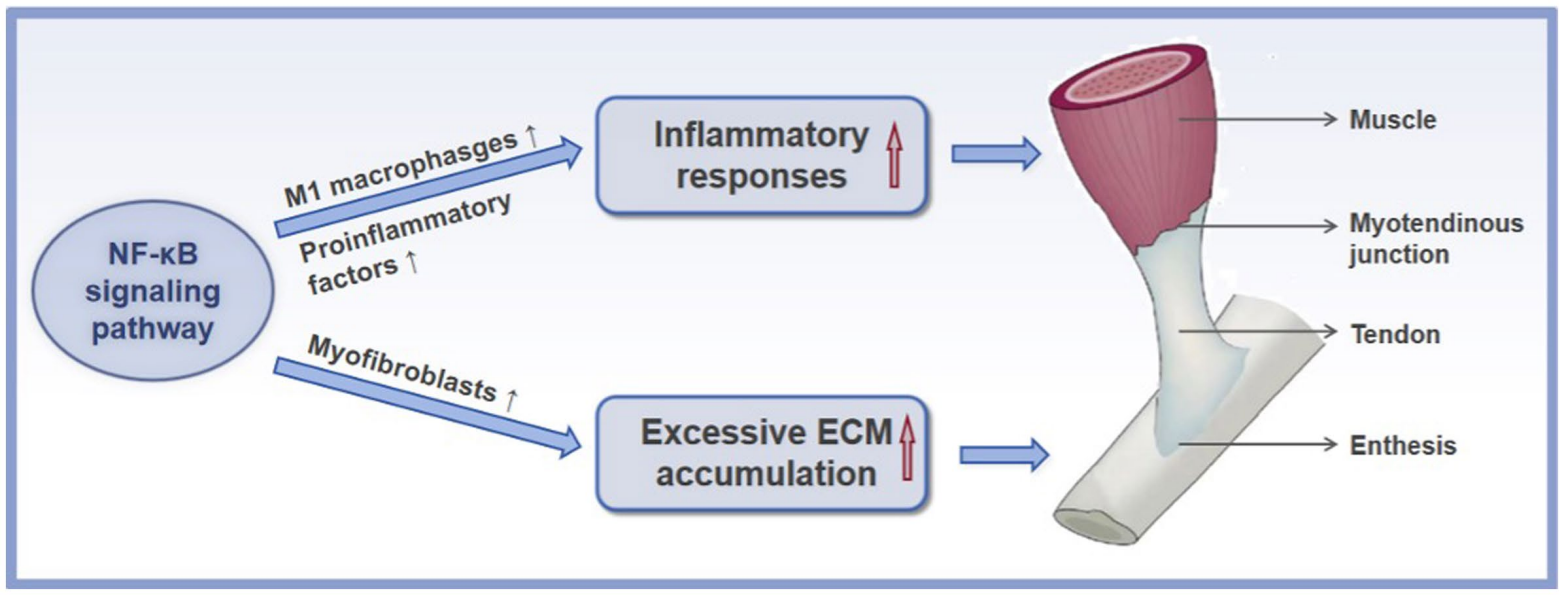
The mechanisms of the NF-κB signaling pathway in the formation of scar and adhesion after acute tendon injury. ↑ refers to upregulation or increase.

As mentioned above, scar tissue formation and tendon adhesion are two primary issues encountered after acute tendon injury. The NF-κB signaling pathway is a key player involved in these processes. Mechanically, the NF-κB signaling pathway promotes the tendon scar and adhesion formation by facilitating the activation of M1 macrophages, promoting the production of proinflammatory factors, accelerating the persistent presence of myofibroblasts, and inducing the ECM accumulation (Figure 3), which will be thoroughly described below.

### 4.1 NF-κB amplifies the inflammatory responses

Although inflammation is an essential step for proper tendon regenerative healing, a marked early inflammatory response often leads to the formation of a scar-like tendon and adhesions, which disturb the intrinsic repair process and result in a tendon with poor tissue quality and inferior mechanical properties (58). Controlling the inflammatory environment after tendon injury is, therefore, a potential therapeutic approach for promoting tendon regenerative healing (59). Regulatory mechanisms in inflammation are triggered by inflammatory cell infiltration and cytokine production. Various cellular and gene regulatory mechanisms modulate both inflammatory cell infiltration and cytokine production. One such mechanism is the activation of the NF-κB signaling pathway. This was confirmed in a canine flexor tendon injury model, whereby inhibition of IKK-β activity and thereby decreased the activation of the NF-κB signaling pathway was demonstrated to reduce the early inflammatory response and decrease the formation of scar and adhesion tissue compared to the saline-treated group (60). Some reports describe the underlying mechanisms of NF-κB signaling pathway in the inflammatory response during fibrotic tendon healing.

The excessive inflammation during tendon scar healing has been identified to be mainly driven by macrophage accumulation (61). In fact, macrophages primarily display two different phenotypes, with polarization into proinflammatory phenotype (M1) during the early tendon healing process followed by switching to an anti-inflammatory phenotype (M2) during the latter tendon healing process (62). The M1 macrophage polarization is thought to trigger the formation of scar tissue and adhesion. The M1 macrophage polarization is also revealed to cause the release of harmful proinflammatory factors, which may further cause injury to the tendon tissue. It is well established that the activation of the NF-κB signaling pathway plays a key role in the macrophage polarization toward M1 macrophages during the healing process of the injured tendon tissue. Dates from healing mouse flexor tendons has demonstrated that overactivation of the NF-κB signaling pathway enhanced the presence of proinflammatory macrophages at the injured area, resulting in elevated formation of scar tissue and disrupting the mechanical properties of the healing tendon (63). Additionally, an experimental study in a rat rotator cuff healing model displayed that inhibiting the NF-κB signaling pathway, deceased macrophage polarization toward M1 macrophages and thereby reduced the production of excessive inflammatory factors and finally provoked the regenerative potential of injured tissue (64). Previous reports have indicated that microRNAs (miRNAs) primarily function as a silencer of coding genes and that the expression of miRNAs is thought to play a significant role in injured tissue’s healing process. In a study by Shen et al. (65), the delivery of miR-147-3p mimic to treat the injured Achilles tendon of mice blocked the activation of the NF-κB signaling pathway in a process that is mediated by the activation of TLR receptor, which subsequently led to the reduced M1 macrophage polarization and showed a significant attenuation in proinflammatory factors, and finally resulting in the improved healing process after injury, highlights the critical role of the NF-κB signaling pathway in the inflammatory process during tendon healing. Similarly, blocking the p65 phosphorylation of the NF-κB signaling pathway suppresses M1 macrophage polarization, significantly alleviating inflammation and peritendinous adhesion and scar tissue formation in a rat model of Achilles tendon injury (66). Furthermore, *in vivo*, both Wang et al. (67) and Li et al. (68) found that mitigating the activation of p65 of the NF-κB signaling pathway inhibited M1 macrophage polarization and thereby effectively reduced proinflammatory factors production during fibrotic tendon healing, resulting in the improved tendon healing process. To sum up, the aforementioned evidence indicates the role of the NF-κB signaling pathway in the formation of scar and adhesion by inducing macrophage polarization toward M1 macrophages. Nevertheless, the interaction between M2 macrophage polarization and the NF-κB signaling pathway is poorly defined. Therefore, further report is required to illuminate the correlation between NF-κB pathway and M2 macrophage polarization in the process of tendon scar healing.

On the other hand, up-regulation of proinflammatory factors occurs as a direct response after acute injury in an independent pathway of M1 macrophages. It has been reported that the NF-κB signaling pathway is directly involved in the synthesis and activity of proinflammatory cytokines, including IL-1β, IL-6, and TNF-α (69). During the inflammatory stage of tendon scar healing, binding these cytokines to their corresponding receptors leads to the activation of the NF-κB signaling pathway (70). Activated NF-κB signaling pathway, in turn, triggers a proinflammatory cytokine storm, resulting in the formation of a positive feedback loop that has the potential to produce chronic and excessive inflammation (71, 72). These findings suggest that the NF-κB signaling pathway is a central medium to enhance the production of proinflammatory cytokines. Indeed, in a rat model of Rotator cuff injury, blocking the activation of the NF-κB signaling pathway through IKKβ small-molecule inhibitor (ACHP) prevented the production of the proinflammatory cytokines, IL-1β, IL-6, and TNF-α, and thereby alleviated the excessive inflammation that occurred in the early stages of rotator cuff tendon-to-bone healing and decreased the formation of scar tissue, finally improved healing results (73). Thus, in light of the critical role of NF-κB in mediating inflammation, the NF-κB signaling pathway could be a key treatment target for attenuating inflammation and inflammation-related processes to enhance tendon regenerative healing. However, there is still limited research conducted for understanding of the NF-κB signaling pathway involved in the inflammatory response of acute tendon injury.

### 4.2 NF-κB induces excessive ECM accumulation

Generally, adhesion and fibrotic scar formation are thought to be healing responses to a tendon injury, in which the balance of tissue repair and excessive ECM accumulation is perturbed, favoring the latter (74). The contribution of the NF-κB signaling pathway to fibrotic tendon healing has also been reported to induce excessive ECM accumulation.

Myofibroblasts, tenocytes expressing α-smooth muscle actin (αSMA), participate decisively in the remodeling phase during healing process, potentially resulting in scar and adhesion formation (75). They initially appear in the proliferative phase to synthesize and contract the ECM to close gaps within injured tendons (76). However, the persistence of myofibroblasts is recognized as a putative driver of fibrovascular scar healing in the tendon tissue due to exuberant and sustained ECM production (77). In the flexor tendon healing, they also lead to tendon adhesions between the injured site and the surrounding tissue, preventing tendon gliding (78). As such, disrupting myofibroblast persistence has been suggested for the treatment of scar and adhesion formation. The NF-κB signaling pathway is considered to be a therapeutic target for inhibiting myofibroblast persistence during fibrotic tendon healing. Previous investigations have demonstrated that constitutively activated NF-κB signaling pathway in myofibroblasts was observed in human tendon scar tissue, suggesting that the NF-κB signaling pathway may participate in the myofibroblast persistence (79). Moreover, global blocking of p65 of the NF-κB signaling pathway or pharmacological inhibiting of p65 was shown to reduce myofibroblast presence, accompanied by decreased ECM production, leading to the reduced formation of scar tissue in a rat model of flexor tendon injury (80). Above all, the NF-κB signaling pathway plays a potential role in myofibroblast persistence during fibrotic tendon healing. However, the precise mechanisms regulating the promoting effect of the NF-κB signaling pathway on myofibroblast persistence have not yet been fully elucidated and understood. Additionally, in one study, the reverse transcription-polymerase chain reaction analysis revealed that a significantly increased expression of type III collagen and type I collagen, along with the activation of the NF-κB signaling pathway after flexor tendon injury. The activation of the NF-κB signaling pathway was particularly proportional to the increased expression of type III collagen and its promoter genes (81). Therefore, one plausible mechanism for the NF-κB signaling pathway-mediated fibrotic healing process is that the NF-κB signaling pathway can directly trigger the excessive production of type III collagen. Further studies are critical to illuminate the relation between the NF-κB signaling pathway collagen production.

## 5 The mechanisms of NF-κB in the degenerative tendinopathy

**Figure 4:**
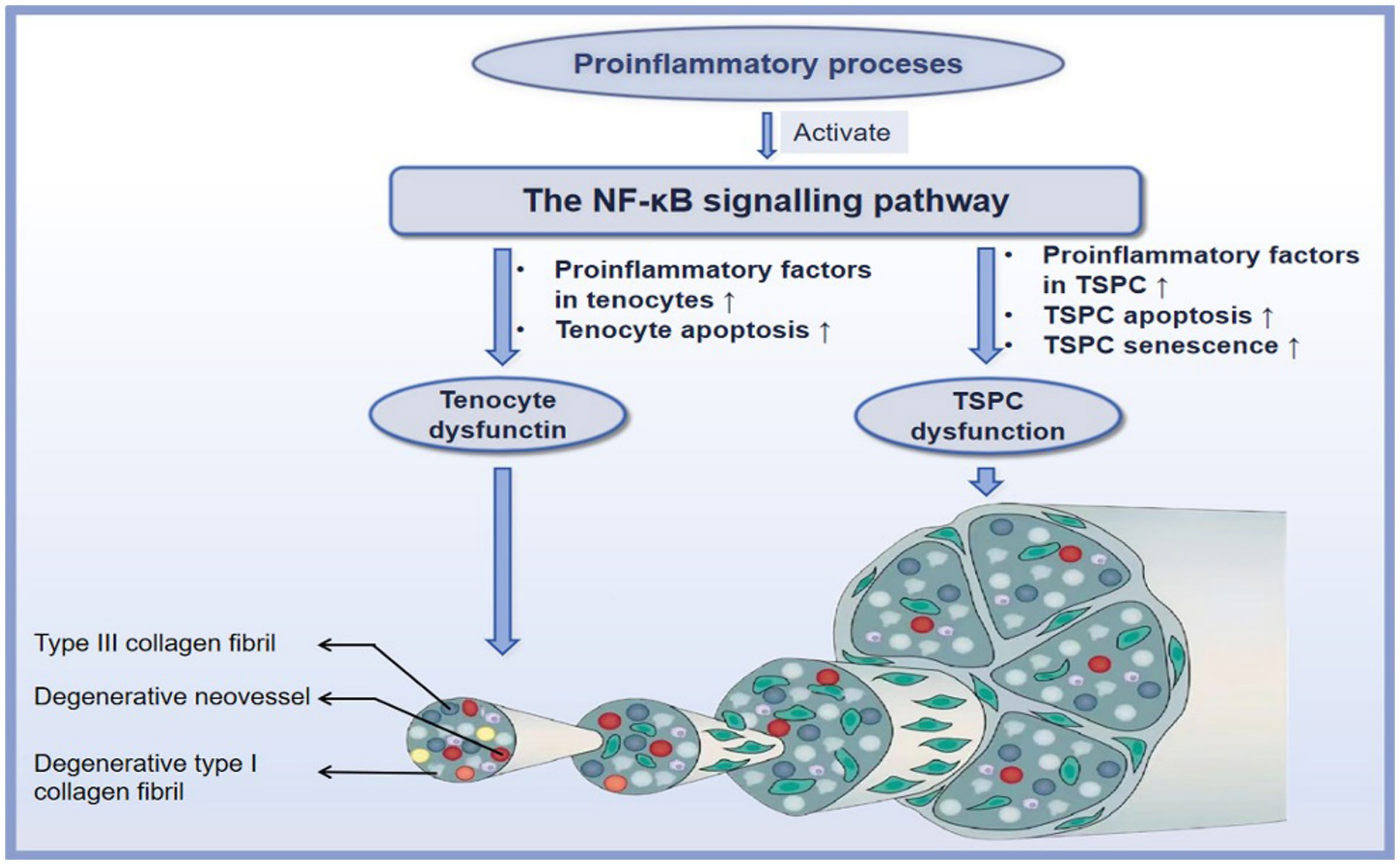
The mechanisms of the NF-κB signaling pathway in the degenerative tendinopathy. ↑ refers to upregulation or increase.

Although the role of inflammation in degenerative tendinopathy has been debated for many years, increasing investigations strongly support that molecular inflammation with elevated inflammatory factors, such as IL-1β, COX2, matrix metalloproteinases (MMPs), and TNF-α, lead to the progression of degenerative tendinopathy (82, 83). The increased expression of these factors initiates a cascade of tendon degeneration, such as ECM destruction, tenocyte apoptosis, TSPCs osteogenesis, senescence, and apoptosis (84). The NF-κB signaling pathway is central to this vicious cycle of degenerative tendinopathy (85). Below, we will thoroughly provide the mechanisms of the NF-κB signaling pathway underlying degenerative tendinopathy (Figure 4).

### 5.1 NF-κB and tenocytes

Tenocytes, the primary local cells of the tendon tissue, exert vital roles in tendon homeostasis. Tenocytes in degenerative tendinopathy have recently been demonstrated to exhibit apoptotic and inflammatory phenotypes. The activation of the NF-κB signaling pathway has been reported to be a critical checkpoint for inflammation-induced degenerative tendinopathy. For example, Buhrmann et al. (86) reported that IL-1β induced the increased expression of COX-2, MMP1, MMP9, and MMP13, and cell apoptosis in human tenocytes and that these mediators resulted in the destruction of tenocytes; subsequently, blocking the activation of the NF-κB signaling pathway can inhibit the detrimental effect of IL-1β. *In vitro*, knockout of IKK-β in tendon fibroblasts from tendinopathic tissue was potentially unresponsive to IL-1β stimulation: results demonstrated decreases in the transcription of MMP-1a, MMP-3, and MMP-13 and in the translation of proinflammatory cytokines and chemokines (87). Furthermore, signaling investigations with specific inhibitors and western blot analysis reported that the underlying mechanisms of IL-1β participate in the above processes through the NF-κB signaling pathway: IL-1β first binds to its receptor IL-1βR and subsequently induces the phosphorylation of Src, and Src being the upstream molecule of Akt and can enhance phosphorylation of Akt. The phosphorylation of Src/Akt directly phosphorylates IKK1 and IKK2, leading to the activation of p65 and p50 of the NF-κB signaling pathway with the consequences for inducing the expression of COX-2 and enhancing the expression of MMP-1, MMP-9, MMP-13 in human tenocytes (88). Conversely, Mueller et al. (89) reported that Calebin A significantly inhibited inflammation-induced MMP-9, COX-2, and cell apoptosis by promoting the expression of SCX and concomitant blocking the p65 of the NF-κB signaling pathway in tenocytes. Moreover, Sirt-1, a nicotinamide adenine dinucleotide (NAD+)-dependent deacetylase, has been demonstrated to be associated with the NF-κB signaling pathway (90). Busch et al. (91), in their *in vitro* investigation, found that Sirt-1 activation inhibited IL-1β-mediated inflammatory factors COX-2, MMP9, and cell apoptosis through inhibiting the activation of the NF-κB signaling pathway in human tenocytes, while down-regulation of Sirt-1 results in similar effects as caused by stimulation with IL-1β. This finding opens the interesting possibility that interfering with NF-κB signaling by activating of Sirt-1 may harbor valuable opportunities for tendon disorders.

### 5.2 NF-κB and tendon stem/progenitor cell

It has long been considered that the majority of cells in tendons are tenocytes, which is well characterized as a regulator of tendon tissue homeostasis and repair. However, a new type of cell residing in the tendons, termed tendon stem/progenitor cell (TSPC), has been identified in horses (92), humans (93), and several other species (94). TSPCs differ from tenocytes in that they can clonogenicity, self-renewal, and multiple differentiation potential. TSPCs are capable of regenerating tendon tissue through tenogenesis, a process of differentiation into new tenocytes (95). Nevertheless, as degenerative tendinopathy progresses, the pathological environment can induce an erroneous differentiation of TSPCs, resulting in chondrocyte-like cells and ossification sites, which are responsible for heterotopic calcification (HO) (96). Furthermore, TSPCs in degenerative tendinopathy also manifest inflammation, cell apoptosis, and senescence. It has been demonstrated that one of the major contributors to TSPC dysfunction is the NF-κB signaling pathway. For example, in cultured TSPCs, inhibition of the NF-κB signaling pathway restored TSPC tenogenic differentiation and effectively decreased the expression of inflammatory factors, including IL-6, MMP-1, MMP-3, and MMP-13 after exposure to IL-1β (97). Moreover, data revealed that the NF-κB and the MAPK signaling pathways exerted a synergistic role in leading to the HO, senescence, and apoptosis of TSPCs after exposure to IL-1β (98). Along with IL-1β, the proinflammatory cytokine TNF-α can also activate the NF-κB signaling pathway and enhance a series of degenerative cascades (99). For example, TNF-α induced inflammation, apoptosis, and HO of TSPCs by activating the NF-κB and MAPK signaling pathways, leading to dysfunction of TSPCs during degenerative tendinopathy (100). These finding suggests that the interaction between NF-κB and other signaling pathways may reveal many potential therapeutic targets to ameliorate the progression of degenerative tendinopathy.

On the other hand, aging is associated with chronic inflammation. During the aging process, a low-grade inflammation network that contributes to the progression of degenerative tendinopathy is activated, termed (inflamm-aging) (101). It has been demonstrated that one of the key contributors to TSPC senescence is inflamm-aging. Once exposed to inflamm-aging, TSPC will be prone to adopt a senescent phenotype and enhance the proinflammatory cytokine milieu, which accelerates the senescence of other cells (102). The NF-κB signaling pathway has been implicated in this process. For example, it has been reported that the senescence progress of TSPCs was remarkably accelerated by the activation of the NF-κB signaling pathway in TSPCs from the degenerative tendinopathy of patients (103). Wang et al. (104) found that target inhibition of the IKKβ can reduce the TSPC senescence response to the inflamm-aging-triggered rat degenerative rotator cuff tendinopathy by inhibiting the expression of the senescence-associated b-galactosidase (SA-b-gal) and cyclin-dependent kinase inhibitor (p21^CIP1A^). Overall, these findings suggest opportunities to reverse the aging process of tendon tissues by targeting the activation of the NF-κB signaling pathway. However, there is still limited studies conducted for understanding its complete mechanisms of action as anti-aging.

Taken together, by mediating the interaction between tenocytes, TSPCs, and inflammatory processes, the NF-κB signaling pathway disturbs tendon homeostasis and induces the progression of degenerative tendinopathy. These studies shed new light on the mechanisms underlying tendinopathy, further establishing the potential of the NF-κB signaling pathway as a therapeutic target for chronic tendinopathy.

## 6 Future prospects of the NF-κB signaling pathway in tendon disorders

Although targeting the activation of the NF-κB signaling pathway has demonstrated promising outcomes in managing tendon disorders, there is still a lot to be explored in this domain.

First, according to investigations, the NF-κB signaling pathway can also interact with other signaling pathways, such as MAPK, to promote the progression of tendinopathy (as also highlighted in Section 5) (105). Also, Fu et al. (106) reported that the NF-κB signaling pathway coordinated with the mTORC1 signaling pathway to induce HO during progression of tendinopathy. On the other hand, Wang et al. (107) found that TGF-β1/Smad3 collaborated with the NF-κB signaling pathway, triggering the tendon scar and adhesion formation following injury. Also, Fu et al. (106) reported that the NF-κB signaling pathway coordinated with the mTORC1 signaling pathway to induce HO during progression of tendinopathy. Considering the interaction of the NF-κB signaling pathway with other signaling pathways that perpetuate the development of tendinopathy, there is a possibility that interfering with NF-κB signaling and these signaling pathways may harbor more promising opportunities for tendon disorders. This possibility remains to be investigated.

Secondly, target blockage of the NF-κB signaling pathway regarding different tendinopathic disease courses is required to be determined. For example, supraspinatus tendon samples from patients with early-stage tendon disorders displayed a mixed inflammation signature with increased expression of genes and proteins mediated by interferon and the NF-κB signaling pathway; intermediate-stage tendon disorders showed an inflammation signature where expression of the NF-κB mediated proinflammatory genes and proteins; advanced-stage tendon disorders showed the increased expression of STAT-6 and glucocorticoid receptor pathway (108). This transition in inflammation activation signatures indicates that therapeutic targeting of the NF-κB signaling pathway may be viable at early and intermediate stages of degenerative tendinopathy. Further investigations to demonstrate the efficacy of targeting the NF-κB signaling pathway at an appropriate time during the progression of tendinopathy are needed.

Lastly, although overwhelming data across the literature indicates that the NF-κB signaling pathway negative affects during tendon healing, another investigation suggests otherwise. For example, blocking IKKβ by gene knockout in the Scx-lineage population was reported to reduced scar tissue and restore tendon failure strength in a mouse model of Rotator Cuff tendon injury (87). In other contradicting studies, it has been reported that deletion of IKKβ in the Scx-lineage population in mice led to enhanced cell apoptosis and ECM accumulation and was not biomechanically beneficial to flexor tendon healing, suggesting that the NF-κB signaling pathway likely plays different roles in a given microenvironment (79). That is, flexor (midsubstance) tendon healing, as used in this study, includes a combination of intrinsic healing and extrinsic healing. Enthesis, as in the supraspinatus tendon, is more complicated by additional cell contributions from the bone, such as osteoblasts and osteoclasts, and the fibrocartilaginous transition region, leading to a different cellular environment. This contradiction likely reflects an incomplete understanding of the action of the NF-κB signaling pathway in mediating tendon healing at different locations. Thus, future studies are warranted to investigate the precise mechanism of the NF-κB signaling pathway in mediated fibrotic tendon healing at different locations.

## 7 Conclusion

Summing up, activation of the NF-κB signaling pathway is associated with the formation of scar and adhesion tissue after acute tendon injury and pathological alternations of chronic degenerative tendinopathy. In terms of mechanisms, after acute tendon injuries, the NF-κB signaling pathway contributes to tendon scar and adhesion by promoting M1 macrophage polarization, inducing production of the proinflammatory cytokines, and enhancing the persistent presence of myofibroblasts. During the progression of degenerative tendinopathy, the NF-κB signaling pathway is a key checkpoint for inflammation-induced degenerative alternations, including promoting tenocyte to produce inflammatory factors, apoptosis and inducing TSPC to produce inflammatory factors, apoptosis, and HO. However, extensive scientific examinations are still warranted to full characterize the NF-κB, the exact mechanisms of action, and translate findings into clinical human and veterinary practice.

## Author contributions

HL: Writing – original draft, Writing – review & editing. SLu: Conceptualization, Data curation, Formal analysis, Funding acquisition, Investigation, Methodology, Project administration, Resources, Software, Supervision, Validation, Visualization, Writing – review & editing. ZF: Writing – original draft, Writing – review & editing. SLi: Writing – original draft, Writing – review & editing. YL: Writing – review & editing. YZ: Writing – review & editing.
